# Person Re-Identification Based on Contour Information Embedding

**DOI:** 10.3390/s23020774

**Published:** 2023-01-10

**Authors:** Hao Chen, Yan Zhao, Shigang Wang

**Affiliations:** College of Communication Engineering, Jilin University, Changchun 130012, China

**Keywords:** person re-identification, pedestrian contour, contour information extraction

## Abstract

Person re-identification (Re-ID) plays an important role in the search for missing people and the tracking of suspects. Person re-identification based on deep learning has made great progress in recent years, and the application of the pedestrian contour feature has also received attention. In the study, we found that pedestrian contour feature is not enough in the representation of CNN. On this basis, in order to improve the recognition performance of Re-ID network, we propose a contour information extraction module (CIEM) and a contour information embedding method, so that the network can focus on more contour information. Our method is competitive in experimental data; the mAP of the dataset Market1501 reached 83.8% and Rank-1 reached 95.1%. The mAP of the DukeMTMC-reID dataset reached 73.5% and Rank-1 reached 86.8%. The experimental results show that adding contour information to the network can improve the recognition rate, and good contour features play an important role in Re-ID research.

## 1. Introduction

Person re-identification (Re-ID), also known as cross-camera pedestrian tracking, aims to identify the same person image under different cameras [1]. Nowadays, face recognition has been widely used in all areas of life, and the research of face recognition has also developed rapidly [2,3,4]. However, in many cases, the camera cannot accurately capture a clear face, so person re-identification technology can play an important role [5].

The initial Re-ID relies on manual features such as color and texture [6,7]. Although it has a certain recognition effect, it still has a certain gap with human recognition. Nowadays, most of Re-ID is based on deep learning, on which researchers are committed to solving various practical problems encountered. Some research is aimed at Re-ID itself, such as solving posture misalignment, image occlusion [8,9], etc. Some research is aimed at deep learning networks [10], such as using attention mechanism, network improvement, small sample recognition [11], etc.

**Figure 1:**
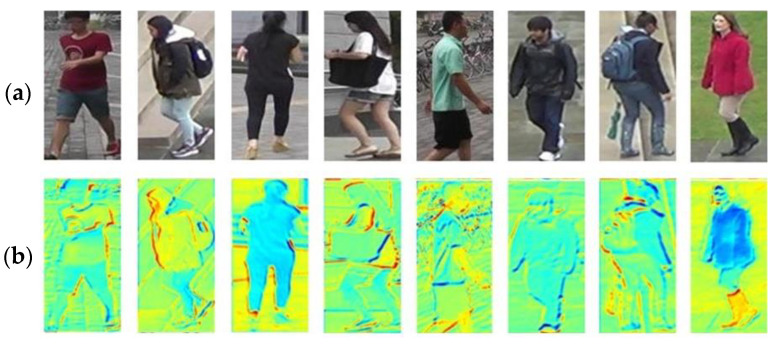
Mapping between original pedestrian images and feature maps (**a**) pedestrian images in common datasets. (**b**) Shallow features of RGB images in ResNet-50 network.

It is found that contour information also plays an important role in image recognition. Some works [12] deeply understand the expression of CNN’s in-depth visual features. Experiments on Imagenet show that CNN-based depth learning models prefer texture-based features to shape-based features [13,14]. In addition, the embedded hybrid model based on texture and contour has been proven to improve the performance of image classification and object detection. We extract the feature map of the ResNet-50 network, which is the most commonly used in Re-ID research, and visualize it (see Figure 1). It can be intuitively found that the CNN network is missing in the extraction of contour information. In the past two years, many researchers began to use contour information as an auxiliary item to participate in Re-ID research, and achieved some success.

Based on the above existing facts, we believe that adding contour information to the CNN network can effectively help them learn more and more robust pedestrian information, and can improve the final recognition effect. In view of the end-to-end secrecy of CNN network, one cannot intervene with a human’s preconceived knowledge. Therefore, we build contour information extraction module with the help of attention thought, so that the CNN network can pay more attention to some contour information instead of losing it in the process of multi-layer convolution. Essentially, the contributions of this paper are as follows:

- We verify the lack of contour information in CNN network in person re-identification research.
- We propose a contour information extraction module, which can make the network pay more attention to the contour information in the pedestrian image without the intervention of human experience.
- The experimental results show that our method has a good effect on Market1501 and DukeMTMC-reID datasets.

## 2. Related Works

### 2.1. Person Re-Identification Based on Attention Mechanism

In recent years, the attention mechanism has been widely used [15,16,17,18]. Its purpose is to enable the network to learn more important things. In Re-ID research, it can effectively solve the problem of posture misalignment, pedestrian image deviation, and partial occlusion. For example, Liu et al. [19] proposed an attention model into the Re-ID task for the first time to dynamically generate attention features to locate different local areas. Zhao et al. [20] proposed an attention model based on CNN, which uses the similarity information of paired human images to learn the part of the body used for matching. Wang et al. [21] proposed an attention model combining hard attention and soft attention, which can simultaneously learn multi-scale local and pixel level feature maps in an end-to-end manner. Sun et al. [22] proposed a local to global multi-scale attention network (LGMANet), which makes full use of context information and spatial attention information to further improve the recognition ability of the birth network. Zhang et al. [23] proposed an effective relationship-aware global attention (RGA) module, which can also be applied to scene segmentation and image segmentation tasks.

### 2.2. Person Re-Identification Based on Contour Information

In recent years, some researchers have applied pedestrian contour to Re-ID research; for example, Chen et al. [24] first attempted to utilize contour explicitly in deep Re-ID models, and proposed contour guidance, which greatly proves the application prospect of pedestrian contour. Yang et al. [25] believed that on the basis that the change of person’s clothing is not strong (strong refers to the gap between winter clothing and summer clothing), the pedestrian contour also has the ability to distinguish personal characteristics. Based on this, the pedestrian contour map is used as the input of feature extraction for cross-dressing person Re-ID, and good results are achieved. Based on the research of Yang et al., Chen et al. [26] combined with the attention mechanism, used the pedestrian contour to carry out the research on cross dressing Re-ID research. Recently released research [27] proposed a multi-scale appearance and contour deep infomax (MAC-DIM) to maximize mutual information between pedestrian color image features and pedestrian contour features, utilizing contour feature learning as regularization to mine more effective shape-aware feature representation from color images.

## 3. Methods

**Figure 2:**
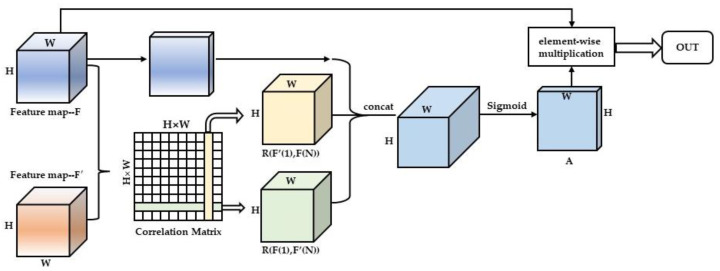
Structure diagram of contour information extraction module. Dimension raising and dimension reducing in the figure are omitted.

**Figure 3:**
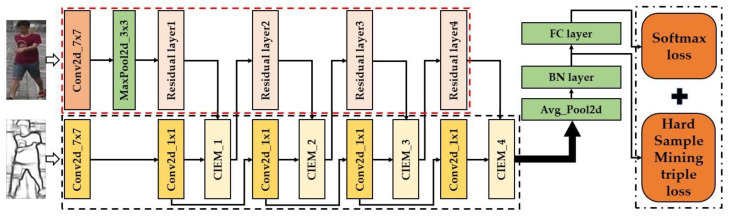
The architecture diagram of the method proposed in this paper.

In this section, we will show the proposed contour information extraction module (CIEM) (as shown in Figure 2), and give a detailed description of the Re-ID method based on contour information embedding (as shown in Figure 3).

### 3.1. Contour Information Extraction Module

The pedestrian contour contains relevant features that are beneficial to learning, but the general CNN network will ignore them to some extent. In order to enable the network to pay more attention to the information contained in the pedestrian contour, we adopt the idea of attention and propose the contour information extraction module. Next, we will introduce the details of the contour information extraction module and how to use it, as shown in Figure 2.

For the pedestrian image in the dataset, when using ResNet-50 for convolution, its output feature map behind a residual layer is set as F, and its size is C × H × W, where C is the number of channels and H × W is the space size. For the pedestrian contour branch, we reduce the dimension of the pedestrian contour map corresponding to the pedestrian image through convolution layer to obtain the contour feature map $F^{\prime }$ with the same size as the feature map F, and its size is 1 × H × W, where 1 is the channel number, H × W is the space size, and F and $F^{\prime }$ are the inputs of the contour information extraction module.

Step 1, reduce the channel number of F to C/8, and increase the channel number of $F^{\prime }$ to C/8 to reduce the network computation.

Step 2, each feature point on the reduced dimension feature graph F is sorted into a one-dimensional vector with the length of *N* = H × W. For convenience, we assign labels from 1 to *N* to each feature point, recorded as $f_{i}\in {\mathbb{R}}^{\frac{C}{8}\times N}$, where i=1,…,N. Similarly, the characteristic points obtained from $F^{\prime }$ are recorded as $f_{j}\in {\mathbb{R}}^{\frac{C}{8}\times N}$, where j=1,…,N.

Step 3, we calculate the correlation matrix $R_{ij}$ between feature map F and contour feature map $F^{\prime }$, the calculation formula is as follows:

(1) $$R_{ij}={\left(f_{i}\right)}^{T}(f_{j})$$

where $R_{ij}\in {\mathbb{R}}^{N\times N}$.

Step 4, split the correlation matrix obtained in the previous step. The dimension of the correlation matrix is *N* × *N*, so the transverse feature vector in the matrix represents the correlation between a feature point in F and the contour feature $F^{\prime }$, which we record as $R\left(F\left(1\right),F^{\prime }\left(N\right)\right)\in {\mathbb{R}}^{N\times H\times W}$. Similarly, the longitudinal feature vector in the matrix represents the correlation between a contour feature point in $F^{\prime }$ and feature F, which we record as $R\left(F^{\prime }\left(1\right),F\left(N\right)\right)\in {\mathbb{R}}^{N\times H\times W}$.

Step 5, we concatenate $R\left(F\left(1\right),F^{\prime }\left(N\right)\right)$ and $R\left(F^{\prime }\left(1\right),c\left(N\right)\right)$ together to obtain the contour information diagram relative to the feature map F. We concatenate this relationship graph with the reduced dimension feature graph F, and then reduce the dimension of the concatenated channel number to 1. After the sigmoid function, we obtain the contour correlation weight matrix $A\in {\mathbb{R}}^{1\times H\times W}$.

Step 6, the output of contour information extraction module is as follows:

(2) $$F_{out}=A*F$$

where F is the feature graph before dimension reduction in step 1, “∗” represents element-wise multiplication, that is, multiplication of elements at corresponding positions in tensor. $F_{out}\in {\mathbb{R}}^{C\times H\times W}$.

### 3.2. Overall Architecture

In the previous section, we introduced the contour information extraction module in detail. Next, we will introduce the Re-ID method based on contour information embedding as a whole. The overall framework we proposed is shown in Figure 3.

The whole frame is divided into two branches, with two inputs respectively. The input of the main branch is the RGB pedestrian original image in the dataset, and the input of the contour branch is the pedestrian contour image corresponding to the RGB original image.

In the main branch, we use ResNet-50 as the backbone network, which is generally divided into five parts, namely, a convolution layer conv1 and four residual layers.

The contour branch is mainly composed of five convolution layers and four contour information extraction modules. A convolution layer with the same conv1 parameter as the convolution layer is placed at the front of the contour branch to perform preliminary dimensionality reduction for the contour map. We add a contour information extraction module at the output position of each residual layer of ResNet-50, and place a convolution layer before the contour information extraction module. The input dimension of the convolution layer is 1, the output dimension is 1, the kernel_ size = 1, and the step size is 2, which is used to reduce the dimension of the contour feature map.

The training method used in our experiment is the same as that used in most Re-ID studies. The loss function used in the experiment is softmax loss and the hard sample mining (trihard loss) [28], which we express as $L_{ID}$ and $L_{T}$, respectively. After backbone receives the characteristic map, it receives the characteristic vector through an average pooling layer. After the vector passes through the BN layer, it calculates the loss function $L_{T}$, and its calculation formula is:

(3) $$L_{T}^{}=\frac{1}{P\times K}\sum_{a\in batch}{\left(\underset{p\in A}{\max}d_{a,p}-\underset{n\in B}{\min}d_{a,n}+\alpha \right)}_{+}$$

where *P* represents the number of person ID in a batch, randomly selecting *K* pictures for each person ID. *a* represents the anchor point, *p* represents the positive sample, and *n* represents the negative sample. *A* is the set of positive samples and *B* is the set of negative samples. $\max d_{a,p}$ represents the most difficult positive sample, $\max d_{a,n}$ represents the most difficult negative sample. *α* means margin and is set to 0.3.

The loss function $L_{ID}$ is calculated by the eigenvector obtained from the BN layer output through the linear layer, the calculation formula is:

(4) $$L_{ID}=-\frac{1}{N}\sum_{i=1}^{N}\log\frac{e^{W_{y_{i}}^{T}f_{i}}}{\sum_{k=1}^{C}e^{W_{k}^{T}f_{i}}}$$

where $f_{i}$ is the *i*-th feature, $W_{k}$ corresponds to a weight vector for class *k*, $y_{i}$ is the corresponding class label, *C* is the number of classes in training dataset, and the size of the mini-batch in the training process is *N*.

Therefore, the final loss function of this method is:

(5) $$L=L_{ID}+L_{T}^{}$$

## 4. Experiments

In this chapter, we will prove the effectiveness of the proposed method from the experimental results. Therefore, we have designed a series of ablation experiments. The proposed model will be tested on Market1501 and DukeMTMC-reID datasets to verify the universality of the method in this paper. Comparing our method with the advanced methods in Re-ID research in recent years, our method still has certain competitiveness.

### 4.1. Datasets and Implementation Details

We selected two datasets that are most commonly used in Re-ID research for experiments. The Market1501 dataset [29] contains 1501 different pedestrian IDs, 751 pedestrian IDs in the training set and 750 pedestrian IDs in the test set, with a total of 32,217 images. The DukeMTMC-reID dataset [30] contains 1812 different pedestrian IDs, 702 pedestrian IDs in the training set and 1110 pedestrian IDs in the test set. Among them, we mainly refer to the ablation experiment with Market1501 and DukeMTMC-reID datasets. In the experiment, we use the RCF model to extract the contour of the dataset and build the pedestrian contour dataset.

Before network training, we will perform data enhancement operations on RGB original images and pedestrian contour images, including random clipping, horizontal flipping, and other common image enhancement operations, and in order to unify different datasets, we adjust the input images to 256×128 pixels. In the training process, we use the Adam optimizer to set the learning rate to $8\times 10^{-4}$, the weight decay rate to $5\times 10^{-4}$, the training cycle to 600, and the batch size to 32. After the training, we did not use methods such as re-ranking to optimize the sorting. In the testing phase, we used the cumulative matching characteristics (CMC) [31] of Rank 1 and mean average precision (mAP) [29] to evaluate the performance, like most of the research on Re-ID.

### 4.2. Ablation Study

#### 4.2.1. Necessity of Contour Information

In two different baseline models, we directly use the pedestrian contour map as the feature map to embed it in the middle layer of the network, and name it the contour embedding method (CEM). These two different baseline models are named baseline1 and baseline2. Baseline1 is a weak baseline model, its backbone network is ResNet-50, and the dimension reduction operation of the last residual layer is reserved. Use the pre-trained parameters on ImageNet before network training. Baseline2 is a strong baseline model. Its backbone network is ResNet-50, which removes the dimension reduction operation of the last residual layer. Before training, the network uses pre-trained parameters that are more suitable for Re-ID research. The specific operation is to embed the pedestrian contour map into the output position of the four residual layers of ResNet-50 in the way of element level addition after dimension reduction through the convolution layer, so as to verify that the CNN network ignores the contour information in the process of extracting image features. Table 1 shows the experimental data of our weaker baseline1, baseline1-CEM, more powerful baseline2, and baseline2-CEM, we can get the following observations:

1. In our research on Re-ID, the CNN network lacks in the extraction of contour features and the expression of contour information. This can be seen from the comparison between baseline1 and baseline1+CEM. Although the final recognition rate is not very high, the effect of adding the contour map is clear. For example, on the Market1501 dataset, baseline1 added with CEM is 0.8% higher on the mAP and 1.3% higher on the Rank-1 than the original;
2. For the powerful baseline 2, perhaps due to the optimization of network pre-trained parameters, the CEM method cannot improve the final recognition rate of the network, and the method of directly using the contour map cannot effectively make the CNN network pay attention to more contour information, it will even reduce the original recognition performance. In order to make the network pay attention to the contour information on the strong baseline, the contour information extraction module is proposed.

#### 4.2.2. Select the Position to Add CIEM

From the characteristics of the existing convolutional neural network, the edge, contour and other feature information contained in an image are all shallow feature expressions, and the visualization results of the feature maps of each layer of CNN network are also the same. Therefore, the following experiments are required to verify where to put the contour information extraction module and how to use it. We will use the contour information extraction module at the output positions of the four residual layers of ResNet-50, and name these four positions L1-L4. The experimental results are shown in Table 2, and we can receive the following observations.

The method of using contour information extraction module is quite different from the speculation before the experiment. From the experimental data, when we only add the contour information extraction module after the first three residual layers, the improvement of the experimental results is not obvious. Taking the Market1501 dataset as an example, the final rank 1 of the contour information extraction module used at L1 and L2 output locations is 94.3%, and the final rank 1 of the contour information extraction module used at L1, L2, and L3 output locations is 94.5%; the recognition effect of these two methods is better than that of the baseline model, but the improvement is not large, and the results are similar. When the contour information extraction module is used after the four residual layers, the final recognition effect of the network is 83.8% on rank1 and 95.1% on mAP, which is significantly improved compared with the former two, and also exceeds the baseline model we use. Therefore, we obtain the final model architecture of this article.

### 4.3. Comparison with the State-of-the-Art

We compare our method with the more classical methods in Re-ID research and some more advanced methods proposed in recent years. Table 3 shows the performance of these methods on two commonly used datasets.

First of all, compared with the baseline model, our method can still improve the final recognition effect on a very powerful baseline model; taking Market1501 dataset as an example, our method is 1.7% higher than the baseline model in mAP and 1.3% higher than the baseline model in Rank-1. Moreover, compared with classical Re-ID algorithms, such as SVDNet, PCB, etc., our method has shown a strong competitive advantage, and has absolute advantages in mAP and Rank-1, two commonly used indicators. Compared with some more advanced methods proposed in recent years, our method has its own advantages to some extent. In the relevant experiments on dataset Market1501, our method has a certain advantage in the indicator Rank-1, for example, compared with BoT, DGNet and other methods, our method still has an advantage of about 0.5%. In terms of the indicator mAP, our method is still comparable to most, but there is still a certain gap compared with DG-Net. On dataset DukeMTMC-reID, the results presented by this method are still the same. Our method has certain advantages over other methods in terms of evaluation index Rank-1, but it is not satisfactory in terms of mAP, which is also the direction of our next research and improvement. For the rest, our method does not use the reranking technology, but compared with other methods using the reranking technology, such as cam and dare, our method still has certain advantages.

## 5. Conclusions

In this paper, we propose a Re-ID method based on contour information embedding. With the idea of attention mechanism and the relationship between CNN feature map and contour map, the contour information extraction module is constructed. We use ResNet-50, which is the most commonly used in Re-ID research, as the backbone network, and use the contour information extraction module in its residual layer output position, so that the network can pay more attention to the contour information in the process of feature extraction. Our method is a breakthrough which attempts to use contour information, and it can still achieve very good recognition effect on a very powerful baseline network. The use of contour information is not limited to this, and we hope to have more research on pedestrian contour in the field of pedestrian recognition.

## Figures and Tables

**Table 1:** The model performance (%) after adding the contour map as a feature supplement on two different baseline models.

Methods	Market1501		DukeMTMC-reID	
	mAP	Rank-1	mAP	Rank-1
Baseline1	74.6	88.5	61.0	76.9
Baseline1-CEM	75.4	89.8	64.4	79.9
Baseline2	82.1	93.8	72.0	84.6
Baseline2-CEM	81.8	93.4	71.7	84.2

**Table 2:** Model performance (%) after adding contour information extraction module at the output position of different residual layers.

Methods	Market1501		DukeMTMC-reID	
	mAP	Rank-1	mAP	Rank-1
L1+CIEM	81.7	93.9	72.2	85.2
L1,L2+CIEM	82.0	94.3	72.6	86.0
L1,L2,L3+CIEM	82.4	94.5	73.4	86.7
L1,L2,L3,L4+CIEM	83.8	95.1	73.5	86.8

**Table 3:** Performance (%) comparisons with the state-of-the-art on Market1501 and DukeMTMC-reID.

	Market1501		DukeMTMC-reID	
Methods	mAP	Rank-1	mAP	Rank-1
Baseline	82.1	93.8	72.0	84.6
SVDNet [32]	62.1	82.3	56.8	76.7
DaRe [33]	74.2	88.5	63.0	79.1
HA-CNN [34]	75.7	91.2	63.8	80.5
DuATM [35]	76.6	91.4	64.6	81.8
Part-Aligned [36]	79.6	91.7	69.3	84.4
PCB [8]	77.4	92.3	66.1	81.8
PCB+RPP [8]	81.6	93.8	69.2	83.3
BoT [37]	85.9	94.5	76.4	86.4
DG-Net [38]	86.0	94.8	74.8	86.6
HPM [39]	82.7	94.2	74.3	86.6
CamStyle(RK) [40]	71.5	89.5	57.6	78.3
DaRe(RK) [33]	82.0	88.3	74.5	80.4
Ours	83.8	95.1	73.5	86.8

## Data Availability

Not applicable.
